# Marine Lectins DlFBL and HddSBL Fused with Soluble Coxsackie-Adenovirus Receptor Facilitate Adenovirus Infection in Cancer Cells BUT Have Different Effects on Cell Survival

**DOI:** 10.3390/md15030073

**Published:** 2017-03-14

**Authors:** Bingbing Wu, Shengsheng Mei, Lianzhen Cui, Zhenzhen Zhao, Jianhong Chen, Tao Wu, Gongchu Li

**Affiliations:** College of Life Sciences, Zhejiang Sci-Tech University, Hangzhou 310018, Zhejiang, China; 13803669864@163.com (B.W.); mss1053280369@163.com (S.M.); clz_2016@yeah.net (L.C.); 17826855065@163.com (Z.Z.); cjh15067149909@163.com (J.C.); wutao0920@163.com (T.W.)

**Keywords:** DlFBL, HddSBL, adenovirus, E2F1

## Abstract

Cancer development and progression are usually associated with glycosylation change, providing prognostic and diagnostic biomarkers, as well as therapeutic targets, for various cancers. In this work, *Dicentrarchus labrax* fucose binding lectin (DlFBL) and *Haliotis discus discus* sialic acid binding lectin (HddSBL) were genetically fused with soluble coxsackie-adenovirus receptor (sCAR), and produced through a bacterial expression system. Results showed that recombinant sCAR-DlFBL not only facilitated adenovirus Ad-EGFP infection in K562/ADR and U87MG cells, but also enhanced the cytotoxicity of adenovirus harboring gene encoding *Pinellia pedatisecta* agglutinin (PPA) or DlFBL (Ad-PPA or Ad-DlFBL) on U87MG cells through inducing apoptosis. Recombinant sCAR-HddSBL facilitated Ad-EGFP infection, but dramatically counteracted the cytotoxicity of both Ad-PPA and Ad-DlFBL in U87MG cells. Further analysis revealed that sCAR-HddSBL, but not sCAR-DlFBL, significantly upregulated transcription factor E2F1 levels in U87MG cells, which might be responsible for the adverse effect of sCAR-HddSBL on Ad-PPA and Ad-DlFBL. Taken together, our data suggested that sCAR-DlFBL could be further developed to redirect therapeutic adenoviruses to infect cancer cells such as U87MG, and the sCAR-lectin fusion proteins for adenoviral retargeting should be carefully examined for possible survival signaling induced by lectins, such as HddSBL.

## 1. Introduction

Lectins, carbohydrate-binding proteins that bind reversibly with mono- or oligosaccharides, have been extensively studied in basic and clinical cancer research, and have shown potential prognostic, diagnostic, and therapeutic values. Lectins have shown potential values for developing therapeutic agents for various cancers. For example, *Maackia amurensis* seed lectin [1], Concanavalin A [2], *Fenneropenaeus indicus* hemolymph fucose binding lectin [3], *Polygonatum cyrtonema* lectin [4], as well as MytiLec [5,6,7] were shown to be cytotoxic to various cancer cells through inducing apoptosis or autophagy. Through adenovirus-mediated gene delivery, the mannose binding plant lectin *Pinellia pedatisecta* agglutinin (PPA), as well as marine lectins, such as galectin *Anguilla japonica* lectin 1, *Haliotis discus discus* sialic acid binding lectin (HddSBL), *Dicentrarchus labrax* fucose binding lectin (DlFBL), and *Strongylocentrotus purpuratus* rhamnose binding lectin, could be exogenously expressed in various cancer cells and led to cancer cell death [8,9,10,11]. PPA delivered through a CD123 retargeted oncolytic adenovirus significantly inhibited in vivo leukemic xenograft growth, suggesting a possible anticancer lectin gene therapy strategy for cancer treatment [12].

Cancer progressions are usually associated with altered glycosylation patterns [13]. Lectins have been developed to form various analytical tools such as lectin microarray, lectin-based immunohistochemistry staining, as well as lectin-based promeotic analysis to investigate glycofiles and biomarkers for a variety of cancers, including aggressive breast cancer [14,15], ovarian cancer [16], pancreatic cancer [17], metastatic colorectal cancer [18], prostate cancer [19], and liver cancer [20,21]. Glyco-biomarkers have been widely utilized in cancer prognosis and diagnosis in the past decades.

Increased fucosylation has been linked to development and progression of prostate cancer [22] and certain subpopulations of pancreatic cancer cells [23]. On the contrary, fucosylation deficiency led to adenocarcinoma in mice [24], and decreased core-fucosylation has been shown to be clinically associated with malignancy of gastric cancer [25]. Meanwhile, increased sialylation was often associated with poor prognosis in cancer patients [13]. A recent study showed that desialylation of cancer cells reduced natural killer cell inhibitory sialic acid-binding Ig-like lectin (Siglec) receptors, and increased natural killer cell activating receptor natural killer group 2D (NKG2D), suggesting a precise sialylation editing method for cancer targeting immune therapy [26].

Previously, we established a bacterial expression system to produce lectin PPA genetically fused with soluble coxsackie-adenovirus receptor (sCAR) [27,28], which successfully re-direct adenovirus to preferentially infect drug resistant leukemic K562/ADR cells and lung cancer H460/5Fu cells [29], suggesting that sCAR-lectin combined with adenoviruses could be developed to form therapeutic vectors to deliver anticancer genes into cancer cells. In this work, DlFBL and HddSBL, lectins specific for fucose and sialic acid, respectively, were genetically fused with sCAR, to form sCAR-DlFBL and sCAR-HddSBL fusion proteins, which were produced through a bacterial expression system and utilized to decorate adenovirus through sCAR-viral fibers interaction. Their capability of bridging adenoviruses to infect cancer cells through lectin-mediated cellular recognition were examined. Furthermore, sCAR-DlFBL and sCAR-HddSBL were further utilized in combination with cytotoxic adenoviruses Ad-PPA and Ad-DlFBL. Their cytotoxicity on cancer cells were investigated.

## 2. Results

### 2.1. The Production of sCAR-DlFBL and sCAR-HddSBL Fusion Proteins

**Figure 1:**
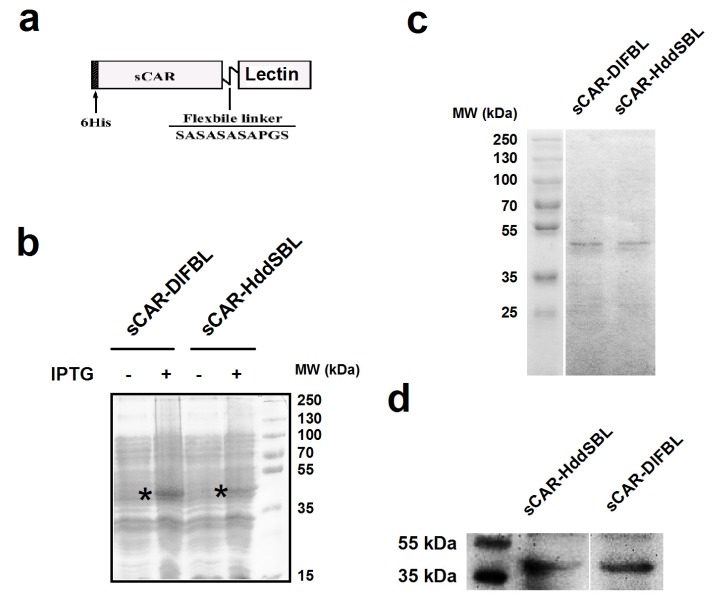
The structure and production of recombinant sCAR-lectin proteins. (**a**) Schematic structure of the sCAR-lectins fusion proteins. The recombinant proteins consist of a 6his-tag, an extracellular domain of CAR with 239 amino acids, a flexible linker (SASASASAPGS), and a lectin region; (**b**) the production of recombinant sCAR-lectin proteins. The pQE30-sCAR-lectin plasmids were transformed to *Escherichia coli* strain M15 and induced by IPTG. The expression of sCAR-DlFBL and sCAR-HddSBL proteins were analyzed by SDS-PAGE followed by Coomassie brilliant blue staining. The sCAR-lectin proteins were purified through a Ni-NTA-Sepharose column, and subjected to SDS-PAGE, followed by Coomassie brilliant blue staining (**c**), and subjected to Western blotting analysis with a goat anti-CAR antibody (**d**).

The sCAR-lectin fusion proteins presented in this work contain a 6his-tag, a human soluble coxsackie-adenovirus receptor (sCAR), a short flexible linker, and a lectin region (Figure 1a). A bacterial expression system was used to produce sCAR-lectin proteins. The production and purification of sCAR-DlFBL and sCAR-HddSBL proteins were examined through SDS-PAGE followed by Coomassie brilliant blue staining (Figure 1b,c). The production of sCAR-DlFBL and sCAR-HddSBL proteins was verified by Western blotting analysis for CAR (Figure 1d). Results indicated that purified sCAR-DlFBL and sCAR-HddSBL with expected molecular weights were successfully obtained through the bacterial expression system.

### 2.2. Recombinant sCAR-DlFBL and sCAR-HddSBL Proteins Facilitated Adenovirus Infection

**Figure 2:**
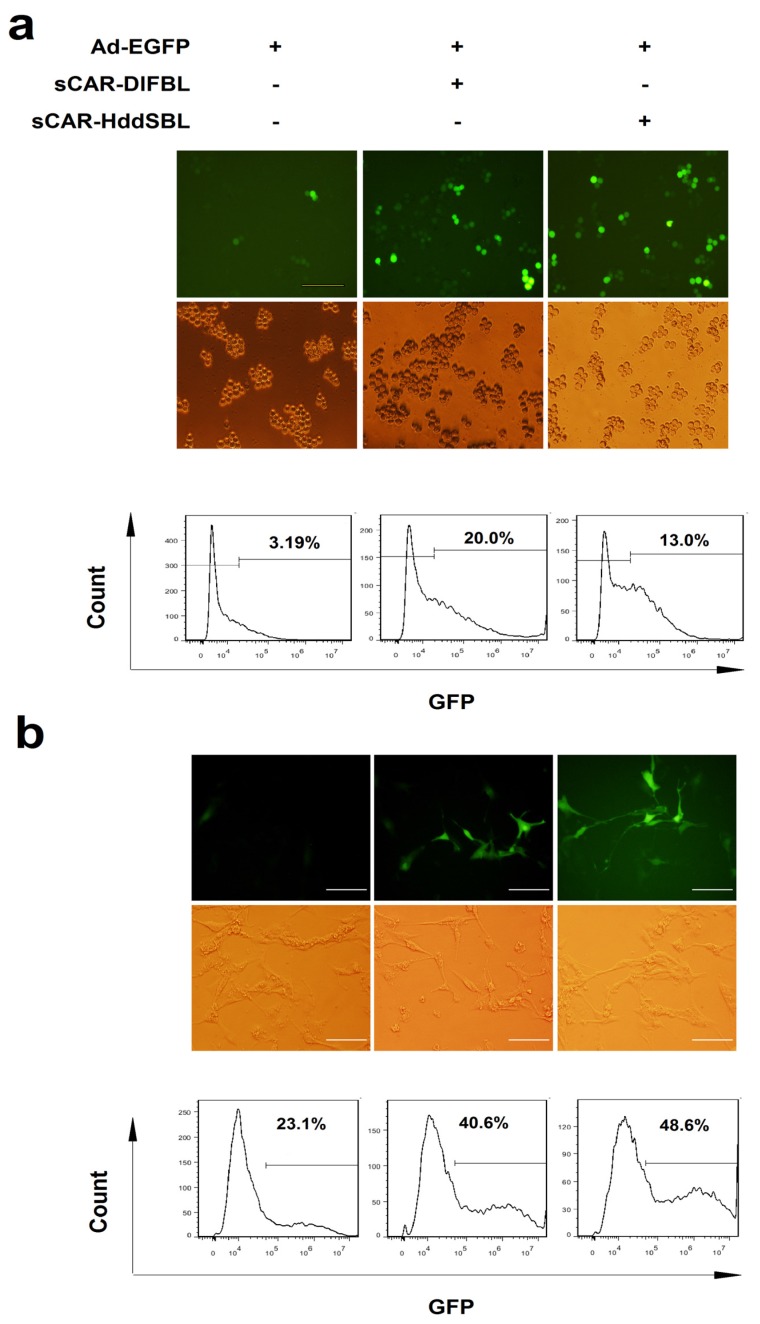
Recombinant sCAR-DlFBL and sCAR-HddSBL proteins enhanced adenoviral infection in K562/ADR leukemia cells and U87MG glioblastoma cells. (**a**) K562/ADR cells were treated with 30 MOI Ad-EGFP combined with 10 μg/mL of sCAR-DlFBL or sCAR-HddSBL for 48 h. Cells treated with Ad-EGFP alone served as the control. The portion of EGFP positive cells was analyzed through fluorescence microscopy and flow cytometry. Shown is a representative from three separate experiments; (**b**) U87MG cells were treated with 5 MOI Ad-EGFP combined with 42 μg/mL sCAR-DlFBL or 31.8 μg/mL sCAR-HddSBL for 48h. Cells treated with Ad-EGFP alone served as the control. EGFP positive cells was analyzed through fluorescence microscope and flow cytometry. Shown is a representative from three separate experiments.

The sCAR-DlFBL and sCAR-HddSBL proteins were then tested for the activity of facilitating adenovirus infection in cancer cells. Leukemic K562/ADR cells were treated with Ad-EGFP alone, as well as Ad-EGFP combined with sCAR-DlFBL or sCAR-HddSBL proteins, followed by fluorescent microscope observation and flow cytometry analysis. As observed under fluorescent microscope, cells treated with Ad-EGFP combined with sCAR-DlFBL or sCAR-HddSBL showed significantly higher portion of EGFP positive population, compared to cells treated with Ad-EGFP alone. Flow cytometry analysis verified the elevated Ad-EGFP infection in K562/ADR cells by sCAR-DlFBL and sCAR-HddSBL proteins (Figure 2a). To further confirm the activity of sCAR-DlFBL and sCAR-HddSBL proteins, the glioblastoma cell line U87MG was infected with Ad-EGFP alone, as well as Ad-EGFP combined with sCAR-DlFBL or sCAR-HddSBL proteins. As shown in Figure 2b, both fluorescent microscope observation and flow cytometry analysis confirmed that sCAR-DlFBL and sCAR-HddSBL proteins were capable of facilitating adenovirus infection.

### 2.3. Recombinant sCAR-DlFBL and sCAR-HddSBL Had Different Effects on the Cytotoxicity of Ad-PPA and Ad-DlFBL in U87MG Cells

**Figure 3:**
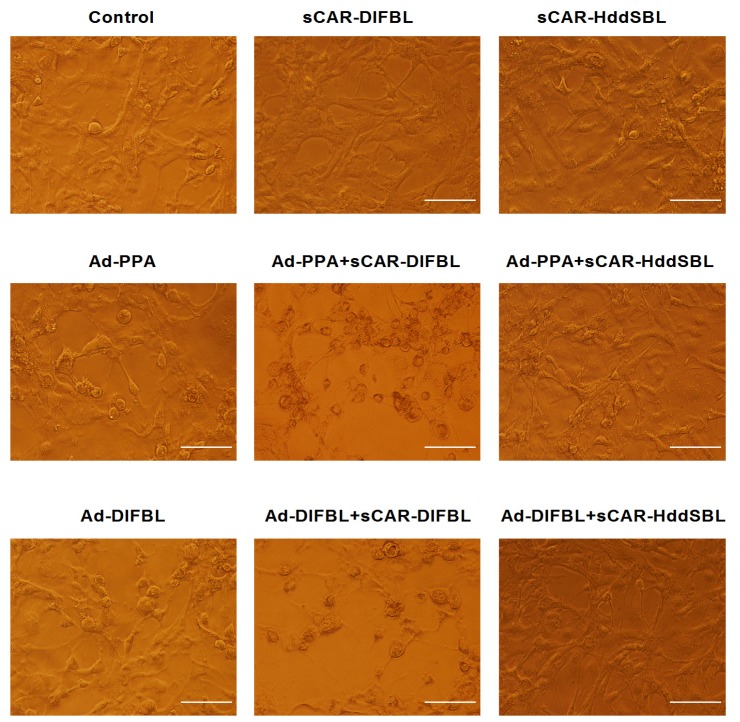
Recombinant sCAR-DlFBL and sCAR-HddSBL had different effects on the cytotoxicity of Ad-PPA and Ad-DlFBL in U87MG cells. U87MG cells were treated with 42 μg/mL sCAR-DlFBL or 31.8 μg/mL sCAR-HddSBL in combination with 6.8 MOI of Ad-PPA or 8.2 MOI of Ad-DlFBL for 48 h. Cells were also treated with PBS, sCAR-DlFBL, sCAR-HddSBL, Ad-PPA, Ad-DlFBL alone as a control. Cell morphology was observed under a microscope.

**Figure 4:**
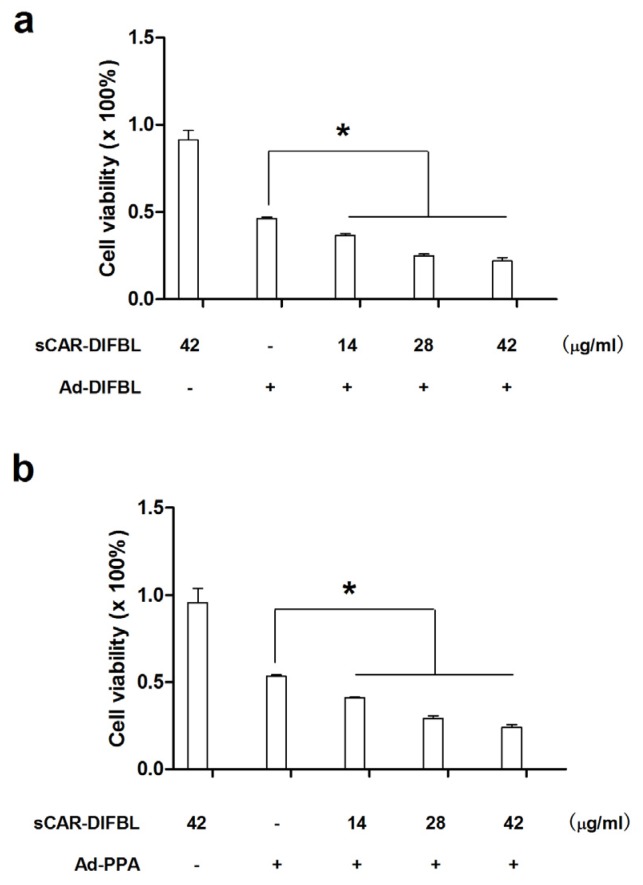
Recombinant sCAR-DlFBL enhanced the cytotoxicity of Ad-PPA and Ad-DlFBL. U87MG cells were treated with (**a**) 8.2 MOI of Ad-DlFBL or (**b**) 6.8 MOI of Ad-PPA in combination with sCAR-DlFBL at concentrations indicated for 96 h. Cells were also treated with PBS, sCAR-DlFBL, Ad-PPA, or Ad-DlFBL alone as a control. Cell viability was analyzed through MTT assay. *: *p* < 0.05

**Figure 5:**
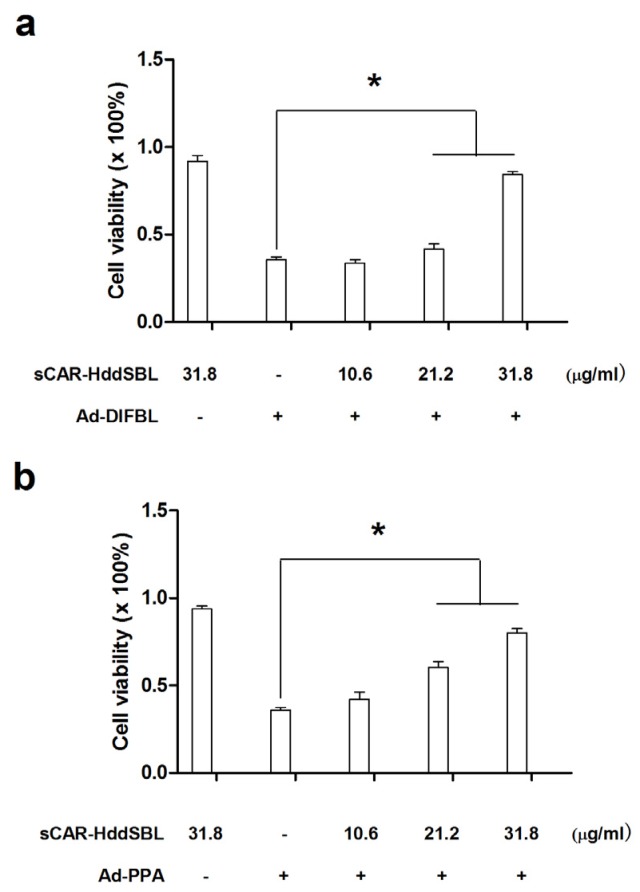
Recombinant sCAR-HddSBL counteracted the cytotoxicity of Ad-PPA and Ad-DlFBL. U87MG cells were treated with (**a**) 8.2 MOI of Ad-DlFBL or (**b**) 6.8 MOI of Ad-PPA in combination with sCAR-HddSBL at concentrations indicated for 96 h. Cells were also treated with PBS, sCAR-HddSBL, Ad-PPA, or Ad-DlFBL alone as a control. Cell viability was analyzed through MTT assay. *: *p* < 0.05

**Figure 6:**
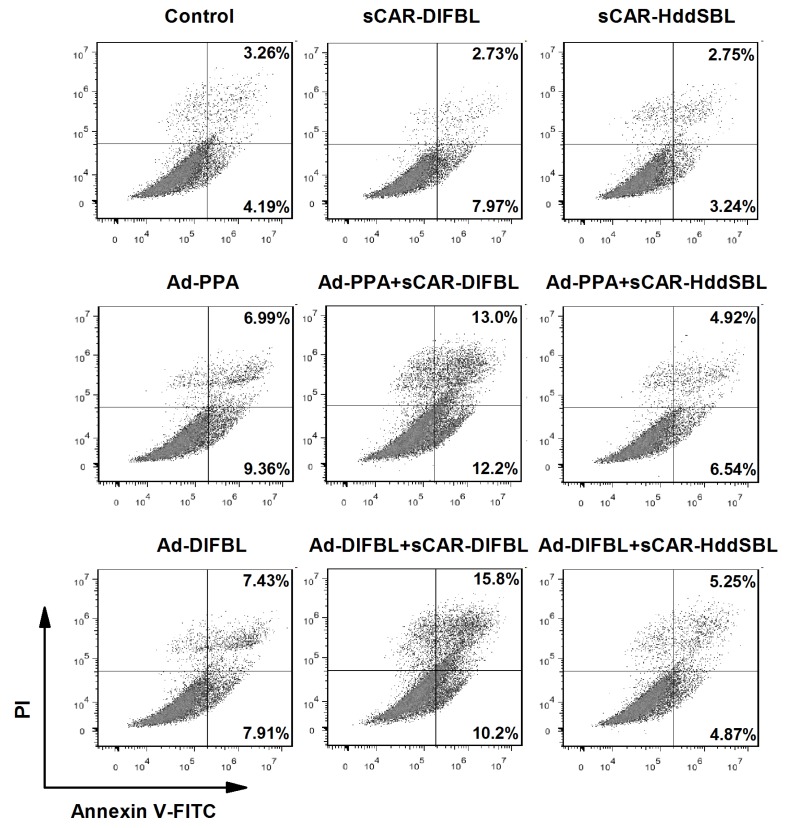
Recombinant sCAR-DlFBL but not sCAR-HddSBL induced apoptosis in combination with Ad-PPA or Ad-DlFBL in U87MG cells. U87MG cells were treated with 42 μg/mL sCAR-DlFBL or 31.8 μg/mL sCAR-HddSBL in combination with 6.8MOI of Ad-PPA or 8.2MOI of Ad-DlFBL for 48 h. Cells were also treated with PBS, sCAR-DlFBL, sCAR-HddSBL, Ad-PPA, and Ad-DlFBL alone as a control. Cells were then collected and stained with by Annexin V-FITC Apoptosis Detection Kit, followed by flow cytometry analysis.

Previously, non-replicating adenovirus harboring gene encoding exogenous lectins, such as PPA and DlFBL have shown significant cytotoxicity to a variety of cancer cells [8,9]. In this work, we tested whether the cytotoxicity of Ad-PPA and Ad-DlFBL could be further enhanced by sCAR-DLFBL or sCAR-HddSBL. As observed under microscope (Figure 3), sCAR-DLFBL in combination with Ad-PPA or Ad-DlFBL dramatically induced higher level of cytotoxicity to U87MG cells than Ad-PPA or Ad-DlFBL alone. Differently, sCAR-HddSBL combined with Ad-PPA or Ad-DlFBL did not induce obvious toxicity to U87MG cells (Figure 3). The higher cytotoxicity of sCAR-DLFBL in combination with Ad-PPA or Ad-DlFBL was confirmed by MTT assay (Figure 4), in which the effect of sCAR-DlFBL showed a dose-dependent tendency. Interestingly, sCAR-HddSBL significantly counteracted the cytotoxicity of Ad-PPA and Ad-DlFBL at a dose-dependent manner (Figure 5). Recombinant sCAR-HddSBL at 31.8 μg/mL almost completely suppressed Ad-DlFBL induced anti-proliferative effect on U87MG cells, suggesting that sCAR-HddSBL possibly activated survival signaling pathways which counteracted the cytotoxicity of Ad-DlFBL and Ad-PPA. Further staining with Annexin V-FITC and PI followed by flow cytometry analysis showed that sCAR-DLFBL but not sCAR-HddSBL in combination of Ad-PPA or Ad-DlFBL enhanced apoptosis in U87MG cells (Figure 6). Our results indicated that sCAR-DlFBL but not sCAR-HddSBL is capable of enhancing the cytotoxicity of Ad-PPA and Ad-DlFBL in U87MG cells through inducing apoptosis.

### 2.4. Recombinant sCAR-HddSBL Upregulated E2F1 Levels in U87MG Cells

**Figure 7:**
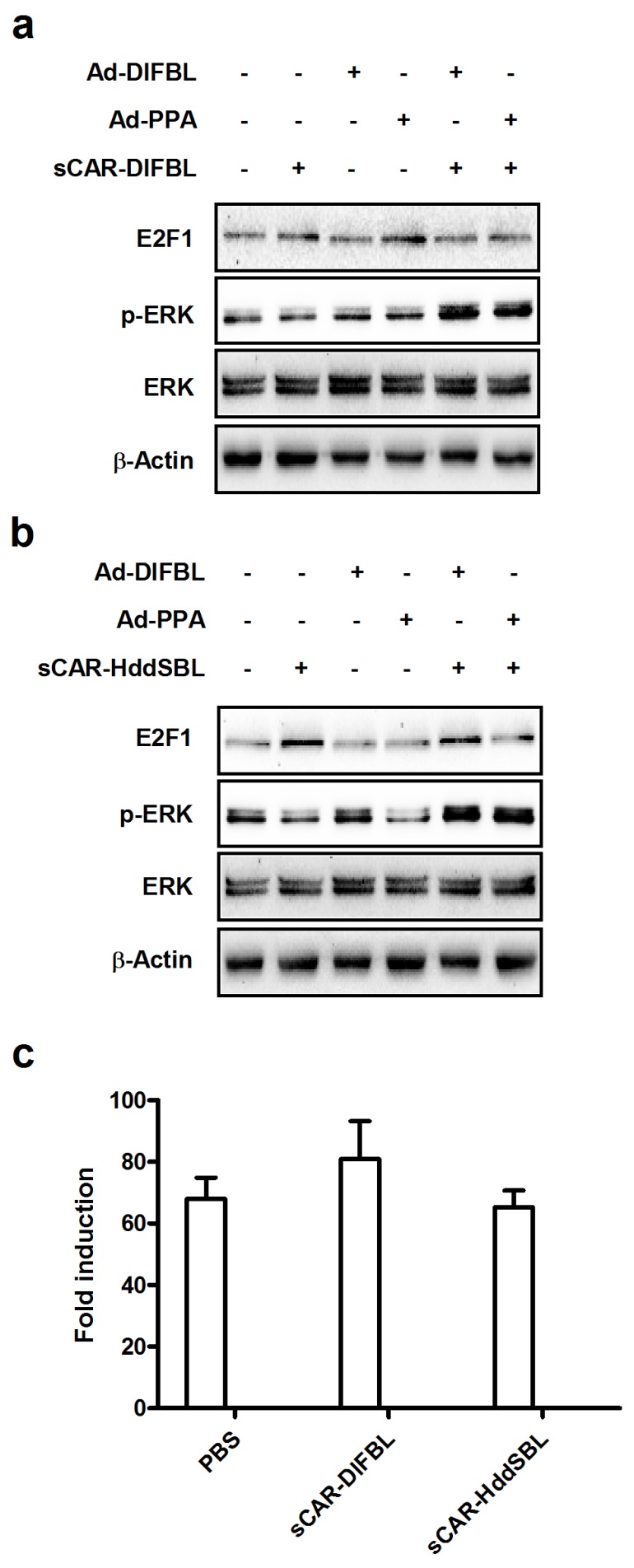
Recombinant sCAR-HddSBL upregulated E2F1 levels in U87MG cells. U87MG cells were treated with (**a**) 42 μg/mL sCAR-DlFBL or (**b**) 31.8 μg/mL sCAR-HddSBL in combination with 6.8MOI of Ad-PPA or 8.2MOI of Ad-DlFBL for 48 h. Cells were also treated with PBS, sCAR-DlFBL, sCAR-HddSBL, Ad-PPA, and Ad-DlFBL alone as a control. Cells were lysed and analyzed for phosphor-ERK, ERK, and E2F1 through Western blot. Actin served as the loading control; and (**c**) the effects of recombinant of sCAR-DlFBL and sCAR-HddSBL on the NF-κB activation in U87MG cells were analyzed through a NF-κB reporter assay.

We then investigated the underlying mechanism of the suppressive effect of sCAR-HddSBL on the cytotoxicity of Ad-DlFBL and Ad-PPA. U87MG cells were treated with sCAR-DlFBL or sCAR-HddSBL in combination with Ad-PPA or Ad-DlFBL as shown in Figure 7a,b. Both sCAR-DlFBL and sCAR-HddSBL combined with Ad-PPA or Ad-DlFBL upregulated the phosphorylation of extracellular regulated protein kinases (ERK), suggesting that ERK was not involved in the sCAR-HddSBL induced suppression effect on the cytotoxicity of Ad-PPA and Ad-DlFBL. Furthermore, NF-κB reporter assay showed that sCAR-DlFBL and sCAR-HddSBL did not significantly alter NF-κB activation (Figure 7c). Interestingly, different from sCAR-DlFBL which had no obvious effect on E2F1 levels (Figure 7a), sCAR-HddSBL alone, or sCAR-HddSBL in combination with Ad-PPA or Ad-DlFBL, significantly upregulated E2F1 levels in U87MG cells as compared to the PBS control, Ad-PPA, or Ad-DlFBL treatment (Figure 7b). Our data indicated that transcription factor E2F1 was significantly upregulated by sCAR-HddSBL.

## 3. Discussion

Due to lytic replication, efficient gene transfer, and low pathogenicity, oncolytic adenovirus, or conditionally replicating adenoviruses, has become a promising strategy for cancer therapy [30,31,32,33]. CAR acts as the primary receptor for the infection of serotype 5 (Ad5) adenoviruses, the most commonly used adenoviral vector in cancer gene therapy [34,35]. However, many tumors only express low levels of CAR, resulting in resistance to Ad5 infection [36]. Previously, we designed a novel strategy to redirect oncolytic adenoviruses to leukemia cell membrane receptors though carrying a sCAR-ligand expression cassette in the viral genome [12,37]. To retarget oncolytic adenoviruses to interleukin-3 receptor α subunit (CD123) or CD47, a sCAR-IL-3, or sCAR-4N1 expression cassettes were genetically inserted into the viral genome. During viral packaging, the sCAR-ligand fusion protein would be expressed in packaging cells and non-covalently installed on viral surface, bridging oncolytic adenoviruses to CD123+ or CD47+ leukemia cells. After infection and replication in leukemia cells, the sCAR-ligand expression would help newly-produced oncolytic adenoviruses to be further modified and infect more leukemia cells. Therefore, harboring a sCAR-ligand expression cassette in the viral genome may become a universal method to redirect oncolytic adenoviruses to various membrane receptors on cancer cells resistant to Ad5 adenovirus infection. In this study, we showed that recombinant sCAR-DlFBL not only facilitated Ad-EGFP infection in K562/ADR leukemia cells and U87MG glioblastoma cells, but also enhanced cytotoxicity of Ad-PPA and Ad-DlFBL in U87MG cells. However, recombinant sCAR-HddSBL enhanced Ad-EGFP infection in U87MG cells, but dramatically counteracted the cytotoxicity of Ad-PPA and Ad-DlFBL. Therefore, our data strongly suggested that sCAR-DlFBL could be further developed to redirect oncolytic adenoviruses to infect cancer cells, such as U87MG. Our data also suggested that sCAR-lectins for adenoviral retargeting should also be carefully examined for possible survival signaling induced by lectins, such as HddSBL in cancer cells.

Transcription factor E2F-1 has been identified as both activator of cell cycle progression and apoptosis inducer [38]. Overexpression of E2F1 was shown to promote leukemia cell proliferation in a cytokine independent manner, and a variety of cell cycle dependant cyclins were maintained by E2F1 without cytokine stimulation [39]. On the other hand, E2F1 induces cell apoptosis through cooperating with either p53 [40] or p73 [41]. In response to DNA damage, Chk2 activates E2F1, which subsequently induces apoptosis [42]. E2F1 was determined to be a direct substrate for PRMT1 and PRMT5 [43,44]. Interestingly, E2F1 methylated by PRMT1 augmented cell apoptosis, whereas E2F1 methylated by PRMT5 favored cell proliferation [44], suggesting a key role of differed arginine methylation in determining E2F1 biological activities. Our previous studies have identified several exogenous lectins such as DlFBL, *Anguilla japonica* lectin 1, as well as *Strongylocentrotus purpuratus* rhamnose binding lectin interacted with PRMT5 and induced downregulation of E2F1 in cancer cells [9,11]. In this work, we further showed that recombinant sCAR-HddSBL upregulated E2F1 in U87MG cells, suggesting that E2F1 may play as a target for various lectins. However, the underlying mechanism is still not clear, pending further investigations.

## 4. Materials and Methods

### 4.1. Production of sCAR-DlFBL and sCAR-HddSBL Fusion Proteins

Plasmid pQE30-sCAR has been constructed previously. In this work, gene encoding *Dicentrarchus labrax* fucose-binding lectin (DIFBL, GenBank accession number: EU877448) or *Haliotis discus discus* sialic acid-binding lectin (HddSBL, GenBank accession No. EF103404) was inserted to form pQE30-sCAR-DlFBL or pQE30-sCAR-HddSBL. The pQE30-sCAR-DlFBL or pQE30-sCAR-HddSBL plasmid was then transformed to *Escherichia coli* strain M15 and the expression was induced by isopropyl β-d-1-thiogalactopyranoside (IPTG). Inclusion bodies were suspended in a buffer containing 8 M urea, 0.1 M sodium phosphate buffer, and 0.01 M Tris-HCl (pH 8.0) at 5 mL per gram of wet weight, and centrifuged at 12,000 rpm for 30 min. The supernatant was diluted by PBS at 1 mL per 20 mL PBS and dialyzed against PBS over night at 4 °C, followed by mixture with Ni-NTA slurry (Merck Biosciences, Darmstadt, Germany). The lysate-Ni-NTA mixture was loaded into a column and washed twice with a washing buffer containing 300 mM NaCl, 50 mM sodium phosphate buffer, and 20 mM imidazole (pH 8.0). The column was eluted with an elution buffer containing 300 mM NaCl, 50 mM sodium phosphate buffer, and 250 mM imidazole (pH 8.0). The eluted protein was dialyzed against PBS at 4 °C overnight to remove imidazole.

### 4.2. Adenoviral Infection

The recombinant serotype 5 adenovirus carrying an enhanced green fluorescent protein gene (Ad-EGFP) was generated in our laboratory previously. K562/ADR cells were treated with 30MOI of Ad-EGFP in combination with PBS or 10 μg/mL of sCAR-lectin proteins. U87MG/Ctr and U87MG/SLMAP cells were treated with 5MOI of Ad-EGFP in combination with PBS or 10 μg/mL of sCAR-lectin proteins. After one day, EGFP positive cells were examined under a fluorescent microscope (Olympus Corporation, Tokyo, Japan) or a BD FACSAria flow cytometry (BD Biosciences, San Jose, CA, USA).

### 4.3. Cytotoxicity Detection

Cells were plated on 96-well plates at 5 × 10^3^ per well one day before infected with adenoviruses. Then cells were treated with adenoviruses in combination with sCAR-lectins as indicated for 96 h. The cytotoxicity detection assay was carried out as the procedure of 3-(4,5-dimethylthiazol-2-yl)-2,5-diphenyltetrazolium bromide (MTT) assays. Meanwhile, cells treated with adenoviruses in combination with sCAR-lectins as indicated for 48 h were collected and stained with Annexin V-FITC Apoptosis Detection Kit (KeyGEN Biotech Co., Ltd., Nanjing, China) following the manufacturer’s instruction, followed by analyzing under a BD FACSAria flow cytometry (BD Biosciences, San Jose, CA, USA).

### 4.4. Western Blot Analysis

The cell extracts were subjected to SDS-PAGE and electroblotted onto nitrocellulose membranes. The membranes were then blocked with Tris-buffered saline and Tween 20 contaning 5% of bovine serum albumin at room temperature for 2 h and incubated with corresponding antibodies overnight at 4 °C. The membranes were washed and incubated with appropriate dilution of secondary antibodies for 1 h at room temperature. After washing with Tris-buffered saline, the bands were detected under a Tanon 5500 chemiluminescence image system (Tanon Inc., Shanghai, China). Prestained protein ladder (Thermo Fisher Scientific, Waltham, MA, USA) and sCAR-lectins bands were detected separately and incorporated together through the software provided by the manufacturer (Tanon Inc.).

E2F1 antibody was purchased from Santa Cruz biotechnology Inc. (Santa Cruz, CA, USA). Phospho-ERK, ERK, and β-Actin antibodies were purchased from Cell Signaling Technology Inc. (Danvers, MA, USA).

### 4.5. Reporter Assay

NF-κB firefly luciferase reporter plasmid was constructed previously. Reporter assay was performed using a duo-luciferase assay kit (GeneCopoeia, Inc., Rockville, MD, USA) following the manufacturer’s instructions. Briefly, U87MG cells were co-transfected with NF-κB luciferase reporter plasmid and Renilla luciferase control plasmid, followed by treatment with PBS, 42 μg/mL of sCAR-DlFBL or sCAR-HddSBL for 24 h. Cells were then lysed and NF-κB firefly luciferase activity was normalized to Renilla luciferase activity.

### 4.6. Statistical Analysis

Differences among the treatment groups were assessed by Student’s *t*-test. *p *< 0.05 was considered significant.

## 5. Conclusions

In this work, recombinant sCAR-DlFBL and sCAR-HddSBL produced in a bacterial expression system successfully facilitated Ad-EGFP infection in K562/ADR and U87MG cells. Recombinant sCAR-DlFBL was further shown to enhance the cytotoxicity of Ad-PPA and Ad-DlFBL on U87MG cells through inducing apoptosis. However, recombinant sCAR-HddSBL dramatically counteracted the cytotoxicity of Ad-PPA and Ad-DlFBL. Further analysis revealed that sCAR-HddSBL upregulated transcription factor E2F1 levels in U87MG cells, which might be responsible for the adverse effect of sCAR-HddSBL on Ad-PPA and Ad-DlFBL. Taken together, our data suggested that sCAR-DlFBL could be further developed to redirect oncolytic adenoviruses to infect cancer cells, such as U87MG, and the sCAR-lectin strategy for adenoviral retargeting should be carefully examined for possible survival signaling induced by lectins, such as HddSBL.
